# Sustained Replication of Synthetic Canine Distemper Virus Defective Genomes *In Vitro* and *In Vivo*

**DOI:** 10.1128/mSphere.00537-21

**Published:** 2021-09-22

**Authors:** Natasha L. Tilston-Lunel, Stephen R. Welch, Sham Nambulli, Rory D. de Vries, Gregory W. Ho, David E. Wentworth, Reed Shabman, Stuart T. Nichol, Christina F. Spiropoulou, Rik L. de Swart, Linda J. Rennick, W. Paul Duprex

**Affiliations:** a Center for Vaccine Research, University of Pittsburgh School of Medicine, Pittsburgh, Pennsylvania, USA; b Department of Microbiology and Molecular Genetics, University of Pittsburgh School of Medicine, Pittsburgh, Pennsylvania, USA; c Department of Microbiology, Boston University School of Medicine, Boston, Massachusetts, USA; d Viral Special Pathogens Branch, Centers for Disease Control and Prevention, Atlanta, Georgia, USA; e Department of Viroscience, Erasmus MC, University Medical Centre Rotterdam, Rotterdam, The Netherlands; f J. Craig Venter Institute, Rockville, Maryland, USA; Icahn School of Medicine at Mount Sinai

**Keywords:** canine distemper virus, defective interfering viral particles, defective interfering genomes, defective genomes, next-generation sequencing, single-step attenuation, vaccines, ferret model

## Abstract

Defective interfering (DI) genomes restrict viral replication and induce type I interferon. Since DI genomes have been proposed as vaccine adjuvants or therapeutic antiviral agents, it is important to understand their generation, delineate their mechanism of action, develop robust production capacities, assess their safety and *in vivo* longevity, and determine their long-term effects. To address this, we generated a recombinant canine distemper virus (rCDV) from an entirely synthetic molecular clone designed using the genomic sequence from a clinical isolate obtained from a free-ranging raccoon with distemper. rCDV was serially passaged *in vitro* to identify DI genomes that naturally arise during rCDV replication. Defective genomes were identified by Sanger and next-generation sequencing techniques, and predominant genomes were synthetically generated and cloned into T7-driven plasmids. Fully encapsidated DI particles (DIPs) were then generated using a rationally attenuated rCDV as a producer virus to drive DI genome replication. We demonstrate that these DIPs interfere with rCDV replication in a dose-dependent manner *in vitro*. Finally, we show sustained replication of a fluorescent DIP in experimentally infected ferrets over a period of 14 days. Most importantly, DIPs were isolated from the lymphoid tissues, which are a major site of CDV replication. Our established pipeline for detection, generation, and assaying DIPs is transferable to highly pathogenic paramyxoviruses and will allow qualitative and quantitative assessment of the therapeutic effects of DIP administration on disease outcome.

**IMPORTANCE** Defective interfering (DI) genomes have long been considered inconvenient artifacts that suppressed viral replication *in vitro*. However, advances in sequencing technologies have led to DI genomes being identified in clinical samples, implicating them in disease progression and outcome. It has been suggested that DI genomes might be harnessed therapeutically. Negative-strand RNA virus research has provided a rich pool of natural DI genomes over many years, and they are probably the best understood *in vitro*. Here, we demonstrate the identification, synthesis, production, and experimental inoculation of novel CDV DI genomes in highly susceptible ferrets. These results provide important evidence that rationally designed and packaged DI genomes can survive the course of a wild-type virus infection.

## INTRODUCTION

Negative-sense (−) RNA viruses are prone to replication errors due to their low-fidelity RNA-dependent RNA polymerase (RdRp). This results in a rich population of genetic variants, including subpopulations of defective interfering (DI) genomes. DI genomes are defined by their ability to disrupt standard genome replication, either directly by competing for resources or indirectly by triggering the interferon (IFN) pathway (1–5). The most common and well-defined DI genomes include the deletion and copyback types (2). During standard genome replication, the incoming (−) RNA is encapsidated by the viral nucleocapsid protein and contains a single genomic promoter (GP). RdRp binds to this GP and synthesizes complementary positive-sense (+) RNA intermediates or antigenomes. The antigenomic promoter (AGP) present on the antigenomic RNA allows RdRp to bind and synthesize novel (−) RNA genomes (6). However, when the RdRp prematurely dissociates from either its (−) or (+) RNA template, it can reinitiate replication by (i) binding downstream on the same template generating deletion genomes or (ii) binding onto the nascent complementary strand generating ambisense genomes known as copybacks. Deletion and copyback genomes that retain functional replication and packaging signals can be maintained alongside the standard genome by utilizing it as a source for missing proteins. This association results in a predator-prey-type scenario which can be visualized as cyclic titer patterns during *in vitro* passage experiments (2, 7).

The ability of DI genomes to disrupt virus replication has led to propositions for their use as vaccine adjuvants or antivirals (7–9). Studies with vesicular stomatitis virus (VSV) (10, 11), Sendai virus (SeV) (12), human respiratory syncytial virus (HRSV) (13, 14), and influenza virus (15–17) have demonstrated reduced standard viral yields both *in vitro* and *in vivo* when DI genomes are present. DI genomes are highly immunostimulatory in nature and demonstrate preferential interaction with RIG-I over the standard genome (18). Copyback DI genomes specifically have been shown to stimulate the production of several proinflammatory cytokines and chemokines and to enhance dendritic cell maturation (13, 19–21). In humans, DI genomes detected in HRSV-positive samples correlate with increased expression levels of antiviral genes (13), while their absence in influenza A virus (IAV)-infected patients correlates with disease severity (22). The antiviral activity of DI genomes has been assessed using both unencapsidated “naked” DI RNA and DI RNA packaged in a ribonucleoprotein complex, defined as a DI particle (DIP). In immunization studies, inactivated viruses adjuvanted with DI RNAs score better than controls with poly(I·C) or alum by inducing type I humoral and cellular immune responses and by enhancing antibody levels (21, 23). This type I IFN-inducing ability allows DIPs to protect against heterologous virus infection, as seen with IAV DIP 244/PR8, which protects mice from unrelated pneumonia virus (24). However, for a DIP-based therapeutic to work, dosage is vital. For instance, mice treated with 400 hemagglutinating units (HAU) (1.2 μg) of IAV DIP 244/PR8 3 weeks prior to a second IAV challenge were completely protected, whereas mice treated with a 10-fold-higher dose did not have the same outcome (15). These results illustrate how achieving a potent dosage optimal for both outcompeting standard virus replication and inducing an immune response is complex.

Another challenge with DIPs is their production. DIPs require appropriate packaging to allow successful delivery of the DI genome of sufficient potency to the right target cells. DI genomes by their very nature are replication deficient and thus need a replication-competent helper virus to drive their production. The inherent enigma here is that the DI genome also interferes with the replication of the helper virus, thereby impeding a straightforward production process. The second challenge is that the DIPs need to then be purified from the helper virus. Certain viruses, like SeV (12), VSV (10, 11), and HRSV (25), exhibit a virion size difference depending on whether the full-length or DI genome is packaged. This allows DIPs to be purified via density ultracentrifugation. This size difference is not seen in all viruses, and separation on the basis of size is not advisable for highly pathogenic viruses. Another option is UV irradiation, which is used to exploit the difference in genome lengths between the helper virus and DIP. At a precise dosage, the much larger full-length genome can be selectively inactivated, while leaving the smaller DI genome intact (8). However, practically, this is not a viable option for highly pathogenic biosafety level 4 (BSL-4) pathogens, for example, Nipah virus. Recently, a packaging cell line expressing the missing IAV protein PB1 demonstrated successful production of pure IAV DIP 244/PR8 without the need for a helper virus (26). This is an ideal scenario, as establishing such packaging cell lines expressing the repertoire of proteins required for many of the other viral DIPs may not be straightforward.

In this study, we investigated a novel, rationally attenuated, recombinant canine distemper virus (rCDV) as a producer virus (i.e., a helper virus) in order to generate rCDV DIPs. CDV is a morbillivirus in the paramyxovirus family, and as with many other members, it requires a polyhexameric genome length for replication (27). Therefore, we focused on rule-of-six-compliant rCDV DI genomes that were generated naturally during *in vitro* passages. We developed assays to generate these rCDV DIPs using our producer virus system and assessed the DIPs for interference activity. Most importantly, we demonstrate that a synthetically engineered fluorescent defective genome can successfully replicate and be maintained in ferrets during the course of a natural rCDV infection. CDV is a tractable BSL-2 pathogen, and ferrets are a naturally susceptible animal model, making our system a good resource for developing highly complex assay systems, such as the ones required for DIPs.

## RESULTS

### A single dominant defective viral genome generated early during *in vitro* rCDV^RI^ infection is consistently maintained across subsequent passages.

To identify predominant DI genomes arising naturally during a CDV infection, we serially passaged four different versions of rCDV strain Rhode Island (rCDV^RI^) 10 times in Vero cells modified to express canine CD150 (Vero-cCD150 cells). First, a plasmid encoding the full-length rCDV^RI^ antigenome was modified to encode rCDVs expressing the reporter proteins Venus, monomeric blue fluorescent protein (TagBFP), dTomato fluorescent protein (dTom), and *Gaussia* luciferase (Gluc) from an additional transcription unit (ATU) at position 6 in the genome. Viruses rCDV^RI^Venus(6), rCDV^RI^TagBFP(6), rCDV^RI^dTom(6), and rCDV^RI^Gluc(6) were generated in Vero-cCD150 cells. Here, cells were first infected with a recombinant vaccinia virus expressing T7 polymerase (MVA-T7) and then transfected with expression plasmids expressing the nucleocapsid (N), phospho- (P), and large (L) proteins, along with the full-length plasmids pCDV^RI^Venus(6), pCDV^RI^TagBFP(6), pCDV^RI^dTom(6), and pCDV^RI^Gluc(6). Virus was rescued 5 to 7 days posttransfection, and a clonal population was generated by plaque-picking syncytia.

**FIG 1:**
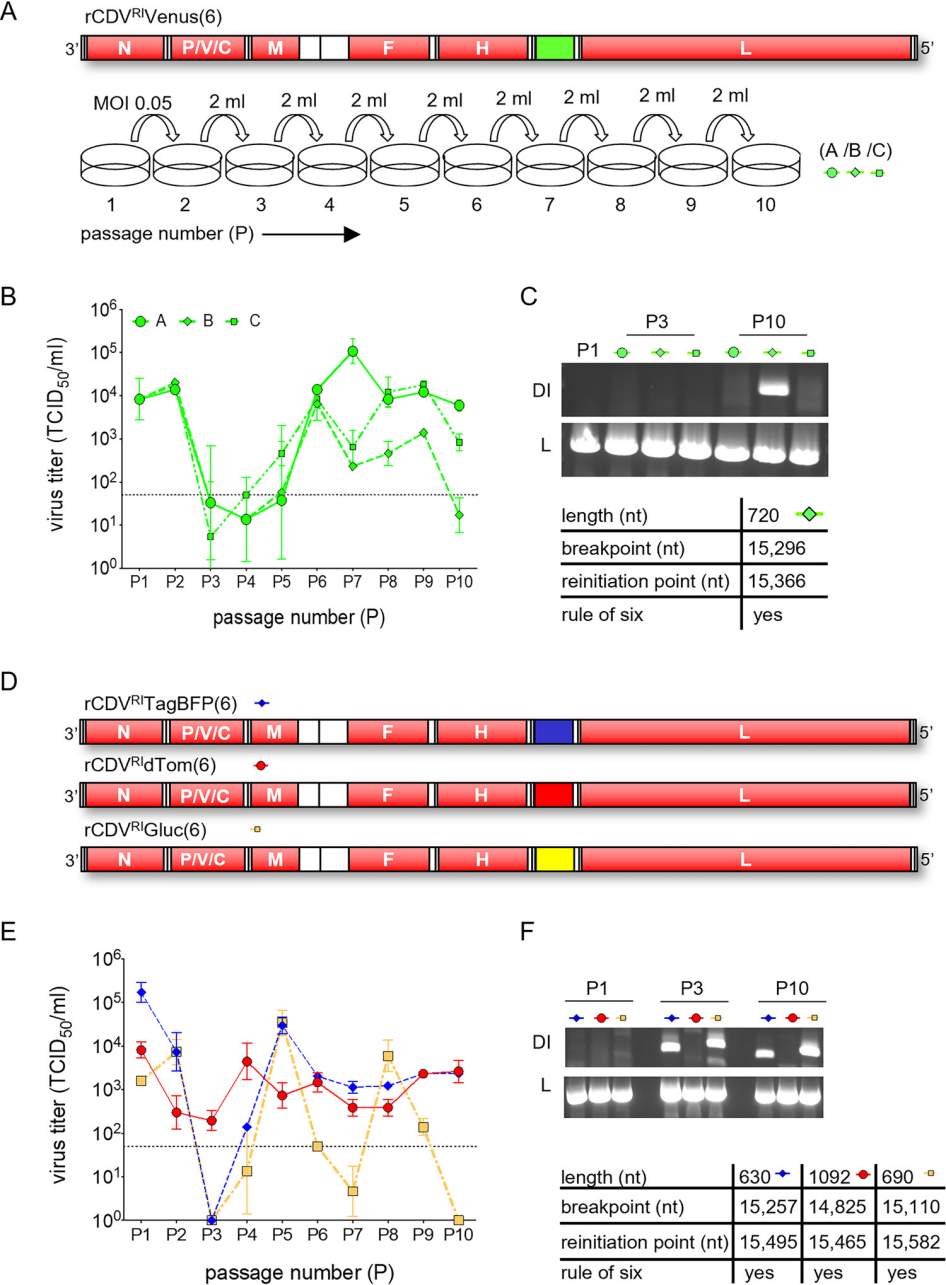
Serial *in vitro* passage of rCDV^RI^. (A) rCDV^RI^Venus(6) was rescued, plaque picked, and passaged once (P1) in Vero-cCD150 cells. Nine serial passages were carried out in a fixed volume (2 ml) in triplicate (A, B, and C). (B) Samples from each passage were titrated in Vero-cCD150 cells and are represented as TCID_50_s per milliliter. Error bars represent standard deviations (*n* = 3), and the dotted line represents the limit of detection for the TCID_50_ assay. (C) RT-PCR was carried out on passages A, B, and C. Copyback-genome-specific primers detected nDI^RI^07cb (length, 720 nt) in passage B at P10. Part of L was amplified as a positive control for viral RNA. (D) rCDV^RI^TagBFP(6), rCDV^RI^dTom(6), and rCDV^RI^Gluc(6) were rescued, plaque picked, and serially passaged 10 times in a fixed volume (2 ml) of Vero-cCD150 cells. (E) Viral titers were determined as described above. (F) RT-PCR using copyback-genome-specific primers amplified different DI genomes for rCDV^RI^TagBFP(6), rCDV^RI^dTom(6), and rCDV^RI^Gluc(6), corresponding to the lengths 630 (nDI^RI^04cb), 690 (nDI^RI^11cb), and 1,092 (nDI^RI^10cb) nt, respectively. Viral RNA was confirmed in all samples by amplifying L.

In the first experiment, rCDV^RI^Venus(6) was passaged once (P1) at a multiplicity of infection (MOI) of 0.05 in Vero-cCD150 cells. This stock was then serially passaged nine times, in triplicate (passages A, B, and C) (Fig. 1A). We found that passages A, B, and C followed highly similar viral titer patterns over the 10 passages (Fig. 1B), which we expect is due to identical DI genomes in all three experiments. Using a copyback genome-specific reverse transcription-PCR (RT-PCR) assay (28) (Table 1) (priCDV^RI^-A1R and priCDV^RI^-A2R), we amplified a 720-nucleotide (nt) copyback genome in passage B at P10 (Fig. 1C). To determine if a similar outcome would arise when repeated, rCDV^RI^TagBFP(6), rCDV^RI^dTom(6), and rCDV^RI^Gluc(6) were serially passaged under similar conditions to rCDV^RI^Venus(6) (Fig. 1D). This time, we observed a different titer pattern for each virus (Fig. 1E). rCDV^RI^TagBFP(6) and rCDV^RI^Gluc(6) titers crashed at passage 3, with rCDV^RI^Gluc(6) crashing also at P7 and P10. Viral titers for rCDV^RI^dTom(6), on the other hand, appeared to remain relatively constant throughout the 10 passages. Here, RT-PCR results revealed a unique copyback genome for each virus: 630 nt in rCDV^RI^TagBFP(6), 1092 nt in rCDV^RI^dTom(6), and 690 nt in rCDV^RI^Gluc(6) (Fig. 1F). Genome lengths of the copyback sequences identified by RT-PCR from these passage experiments are compliant with the rule-of-six requirement for morbilliviruses.

**TABLE 1:** Oligonucleotides used in this study^*a*^

Oligonucleotide name	Sequence (5′–3′)	Genome position (nt)	Purpose
priCDV^RI^-A1R	GACAAAGCTGGGTATGATAACT	15665–15686	Copyback/control
priCDV^RI^-A2R	TAAATCGAAAATTATGTGGTTG	15407–15428	Copyback
priCDV^RI^-B1F	CTACCTCGTTTTTACTGGTCTT	14894–14915	Control
priCDV^RI^TaqF	GCATTATAAAAAAACTAAGGATCCAGG	8991–9017	Full-length (qRT-PCR)
priCDV^RI^TaqR	GGACTATCTAGATGGACCTCAG	9064–9085	
CDV^RI^probe	TTCCAGTCATGGACTCTGTTTCAGTGAA	9022–9049	
priDI^RI^04cbTaqF	GTATGCATGGAACATTCCTTGTG	1528–15259^*b*^	nDI^RI^04cb (qRT-PCR)
priDI^RI^04cbTaqR	GGTTAGGAGCCAGATCAACAT	15586–15606	
DI^RI^04cbprobe	TATAGTGCACTGATTAGAAACCACTGA	15558–15584	
priDI^RI^LedTomDelTaqF	GTGGAGTTCAAGACCATCTACA	NA	sDI^RI^dTomdel (qRT-PCR)
priDI^RI^LedTomDelTaqR	CGTTGTGGGAGGTGATGT	NA	
DI^RI^LedTomDelprobe	CCGGCTACTACTACGTGGACACCA	NA	

a F, forward; R, reverse; NA, not applicable.

b Binds in the reverse direction on the full-length genome.

**FIG 2:**
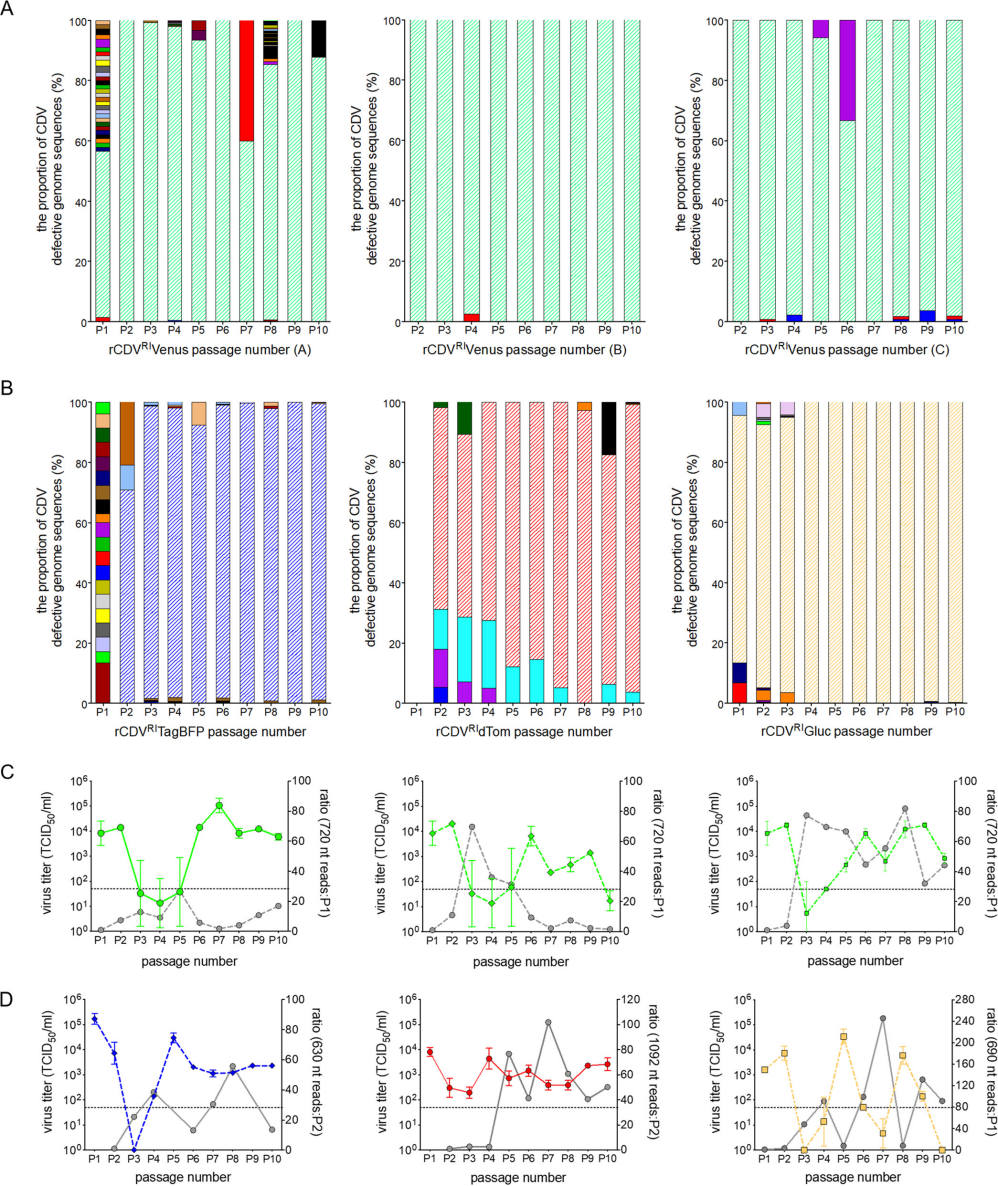
Defective rCDV^RI^ genomes at each passage. (A and B) Relative frequencies of every defective CDV genome at each passage. Each genome is represented by a different color. A read cutoff value of 1 was applied to eliminate sequence noise. (C and D) Sequence reads for the predominant DI genome from panels A and B laid over the viral titers from their corresponding passage experiment. Ratios of reads were calculated from when the predominant DI genome first appeared in a passage; i.e., the diagonal striped areas in panels A and B represent the predominant DI genome. The dotted lines in panels C and D represent the limit of detection for the TCID_50_ assay.

Next, all passaged samples were sequenced using the Illumina MiSeq platform. Data sets were first quality filtered and aligned to their respective reference genome using CLC Genomics Workbench. Data sets were then examined to identify chimeric reads, which consist of either deletion junctions or head-to-tail rearrangements of the genome. These chimeric points were subsequently mapped onto full-length reference genomes, and break and reinitiation points (i.e., the sequence where the RdRp dissociates from the RNA template and the sequence where the RdRp reinitiates replication) were identified. To eliminate false positives that may have passed through the initial screening, we applied a read cutoff value of 1. Using this data set, the frequencies of every defective genome identified across the passages for each virus were plotted. This revealed that each passage contained one predominant defective genome (Fig. 2A and B). Mapping information identified that these copyback genomes were identical to those amplified by RT-PCR, providing confidence in our chimeric Illumina data set.

In the first experiment, using rCDV^RI^Venus(6), a 720-nt genome was identified at P1, and even though passages A, B, and C were independent from P2 onwards, the 720-nt genome remained predominant throughout the nine passages (Fig. 2A). In the second experiment, a 630-nt genome was identified at P2 in rCDV^RI^TagBFP(6) and remained predominant until P10 (Fig. 2B). Whether this genome formed at P1 but was just undetected is unclear. In rCDV^RI^dTom(6), no defective genomes were detected at P1; from P2 onwards, a 1,092-nt copyback genome became predominant. With rCDV^RI^Gluc(6), a 690-nt copyback genome prevailed from P1 until P10 (Fig. 2B). A ratio of the 720-nt, 630-nt, 690-nt, and 1,092-nt copyback genome sequencing reads was calculated for each passage based on when they were first detected. Overlaying these numbers onto their respective parental virus titers demonstrated an inverse correlation in cyclic patterns of DI genome reads and virus titer (Fig. 2C and D). This was especially convincing for rCDV^RI^Gluc(6) (Fig. 2D).

**FIG 3:**
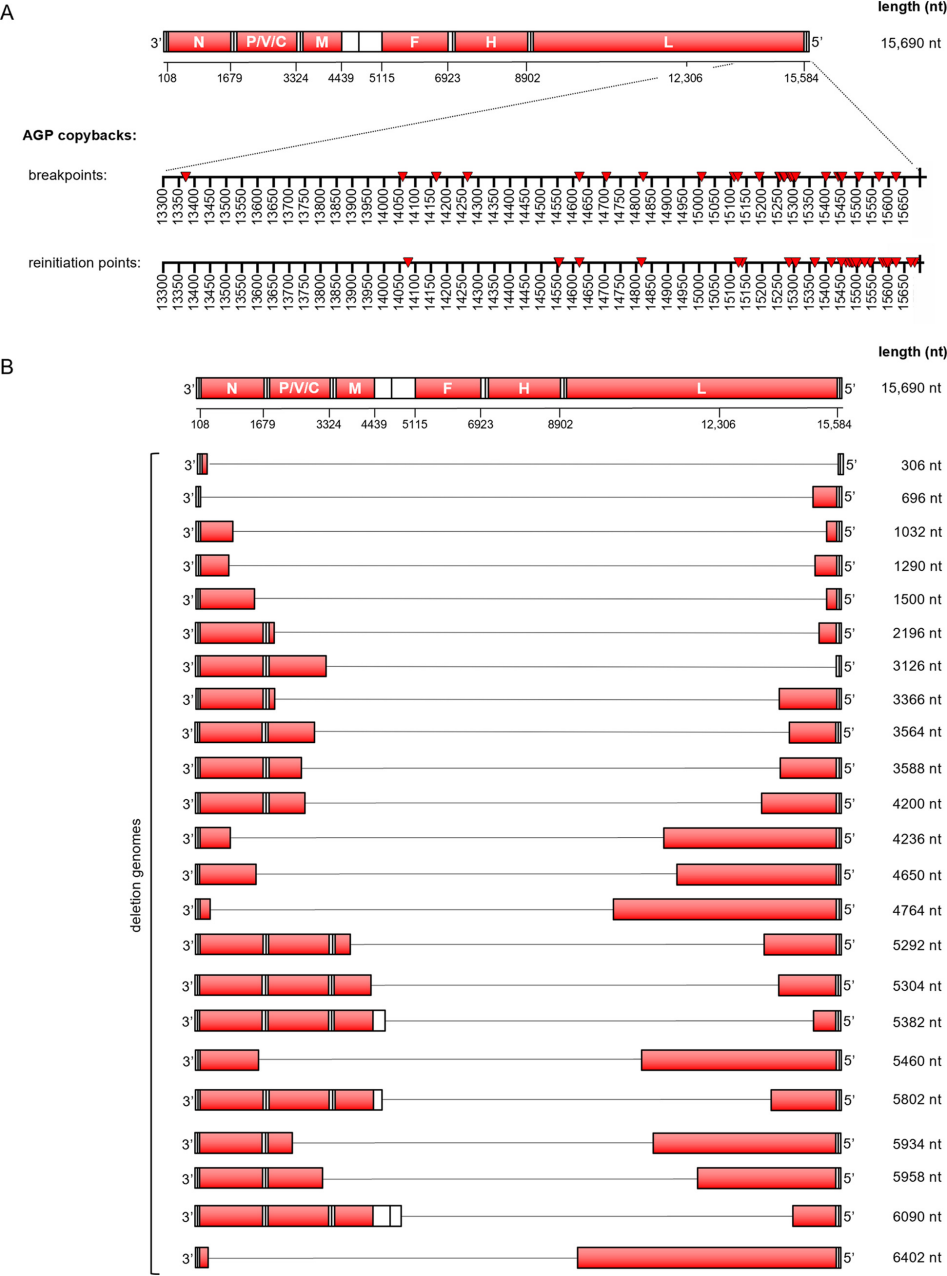
Defective rCDV^RI^ genome break and reinitiation points. (A) Breakpoints and reinitiation points for AGP copyback genomes identified in the passage experiments (Fig. 1B and E) are plotted onto a scale illustrating their location on rCDV^RI^. (B) All rule-of-six-compliant deletion genomes identified in the passage experiments (Fig. 1B and E).

In brief, we identified 15 rule-of-six-compliant rCDV copyback genomes from the serial passages and an additional six during the course of this study (Table 2). We found only four copyback genomes that would have formed toward the GP end of the genome (i.e., GP copybacks). Breakpoints for these GP copyback genomes fell in the N and F genes and were not rule-of-six compliant. Plotting all AGP copyback break and reinitiation points, whether or not these genomes were rule-of-six compliant, we found that breakpoints in our data set typically fell between nt 13371 and 15624 of the rCDV^RI^ genome and clustered between nt 15250 and 15450 (L gene). Reinitiation points for these genomes as expected occurred much closer to the genome end, between nt 15300 and 15683 (Fig. 3A). We found two snap-back genomes (with identical breakpoint and reinitiation sites), one of which was rule-of-six compliant (2,124 nt) (Table 2). In terms of defective genome uniqueness, we found more rule-of-six deletion genomes than copyback genomes (Fig. 3B). However, a majority of these fell below our read cutoff threshold of 1. It is unclear at this time whether these genomes are sequencing artifacts or true deletion sequences that were simply unable to compete with the predominant copyback genome.

**TABLE 2:** Defective rule-of-six-complaint CDV trailer copyback genomes

Genome	Genome length (nt)	Breakpoint (nt position)	Reinitiation (nt position)	Overlap^*a*^	Stem length (nt)^*b*^	Loop length (nt)^*b*^	Detection method^*c*^	Name^*d*^
1	324	15454	15602	2	89	148	NGS	
2	480	15306	15594	2	97	288	NGS	
3	486	15441	15451	4	240	10	NGS	
4	504^*e*^	15332	15546	0	145	124	RT-PCR	
5	528^*e*^	15331	15523	0	168	192	RT-PCR	
6	630	15255	15495	2	196	240	RT-PCR, NGS	nDI^RI^04cb
7	690	15110	15582	0	109	472	RT-PCR, NGS	nDI^RI^11cb
8	720	15295	15366	1	325	71	RT-PCR, NGS	nDI^RI^07cb
9	786	15289	15306	1	385	17	NGS	
10	792	15008	15582	0	109	574	NGS	
11	816^*e*^	14969	15597	0	94	628	RT-PCR	
12	906^*e*^	14913	15563	0	128	650	RT-PCR	
13	936^*e*^	14933	15454	0	237	461	RT-PCR	
14	1,092	14825	15465	0	226	640	RT-PCR, NGS	nDI^RI^10cb
15	1,134	15123	15125	0	566	2	NGS	
16	1,254	14706	15419	3	272	713	NGS	
17	1,422	14823	15136	0	555	313	NGS	
18	1,554^*e*^	14733	15095	0	596	362	RT-PCR	
19	1,734	14166	15482	0	209	1,316	NGS	
20	2,124^*f*^	14620	14620	0	1,071	0	NGS	
21	2,298	14266	14817	0	874	551	NGS	

a Overlap, the number of nucleotides at the breakpoint that may come from either the positive- or the negative-sense strand.

b This number is ± the overlap.

c NGS, next-generation sequencing.

d Named in the order in which they were identified.

e cbDI genome identified from an rCDV^RI^Venus(6) lab stock.

f Snap-back genome.

### Generation of DIPs using rationally attenuated rCDV^RI^ as a producer virus.

**FIG 4:**
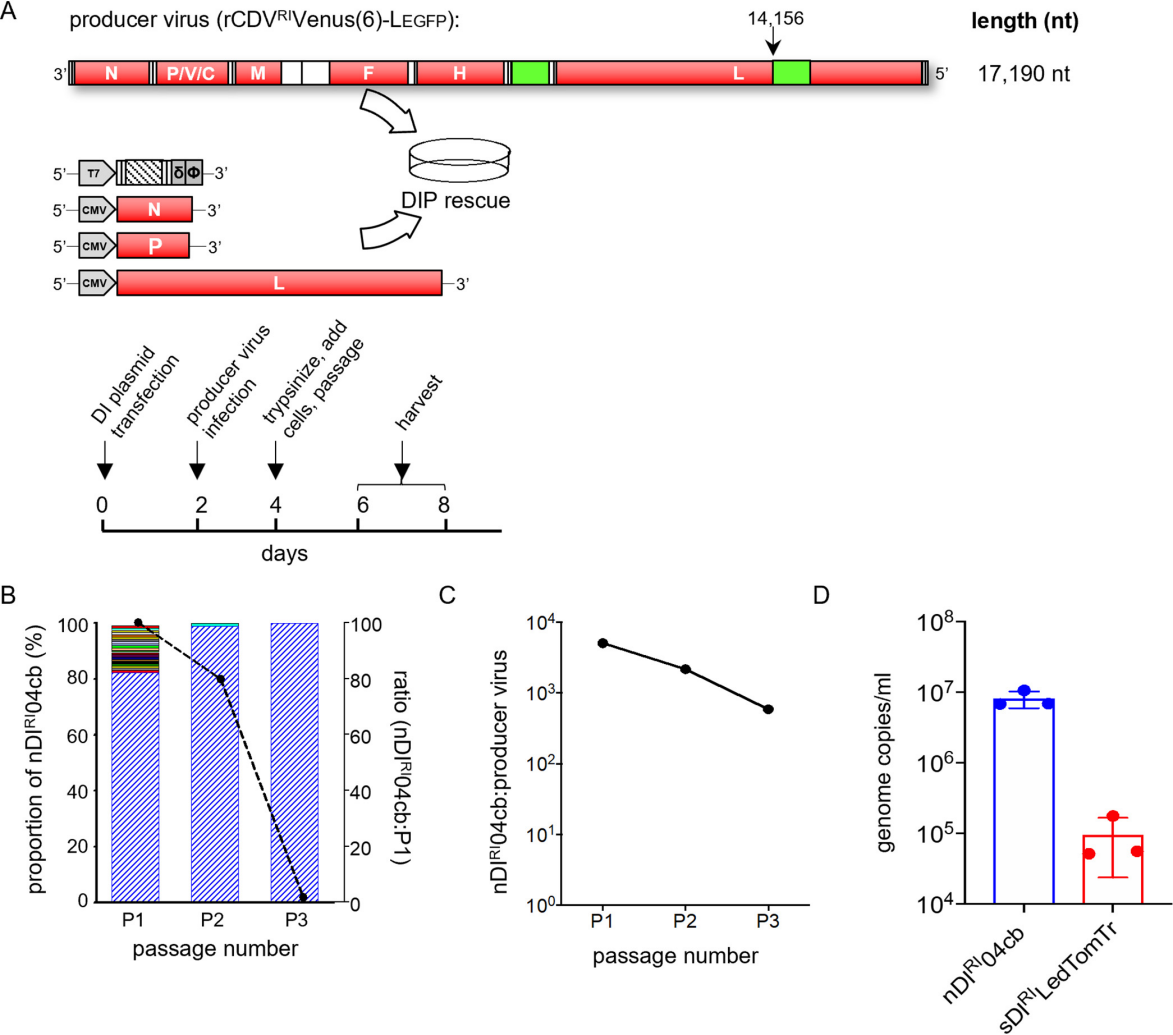
Production of rCDV^RI^ DIPs using a recombinant producer virus. (A) Experimental setup of an rCDV^RI^ DIP rescue. (B) Frequency of defective rCDV^RI^ genomes. Rescued DIP stock still including the producer virus was passaged three times in Vero-cCD150 cells. Blue bars represent the predominant nDI^RI^04cb. The overlaid line graph represents sequence reads for the three passages. (C) The qRT-PCR for the results in panel B shows the ratio of DI to full-length genomes at three passages. (D) Controlling DIP production. qRT-PCR for a passage 2 experiment where a determined ratio of DI to full-length genomes was supplied from passage 1. The experiment was performed in triplicate. Error bars represent standard deviations of titers (*n* = 3).

To produce DIPs in a nonpathogenic background, we generated attenuated rCDV^RI^Venus(6) using an approach described in our previous work (29, 30). We predicted the second variable hinge in the CDV^RI^ L gene to be between nucleotides 14111 and 14180. We mutated the genome positions 14826-27 and 14835-36 in plasmid pCDV^RI^Venus(6) from CT to GG and AA, respectively. This created restriction sites MscI and HpaI, which were used to clone the open reading frame of enhanced green fluorescent protein (EGFP) into the L gene (Fig. 4A). rCDV^RI^Venus(6)-L_EGFP_ was rescued and plaque picked (P0) in Vero-cCD150 cells, with titers similar to those of rCDV^RI^Venus(6). This virus thus encoded both Venus (from an ATU) and EGFP (fused to the viral polymerase). In previous studies, we demonstrated that this fusion protein remains functional as a polymerase, but with reduced efficacy, thus explaining the viral attenuation phenotype.

Next, we investigated whether a clonal population of DIPs can be produced using rCDV^RI^Venus(6)-L_EGFP_. We first constructed DI genome-expressing plasmids by inserting synthetic copyback (cb) DI genome sequences for natural DI^RI^04cb (nDI^RI^04cb), nDI^RI^07cb, nDI^RI^10cb, and nDI^RI^11cb (Table 2) into a T7-driven plasmid backbone. We also constructed a deletion genome (sDI^RI^LedTomTr) by placing the dTom sequence between the leader and trailer sequences of the rCDV^RI^ genome. To then generate DIPs, we transfected Vero-cCD150 cells (supplemented with T7 polymerase) with a DI plasmid and CMV-driven helper plasmids expressing the rCDV^RI^ N, P, and L proteins. Two days posttransfection, these cells were superinfected with rCDV^RI^Venus(6)-L_EGFP_ at an MOI of 0.001, and 6 to 8 days later stocks containing DIPs were generated (Fig. 4A).

To determine the defective genome population in rescued (P0) stocks, we passaged DIP nDI^RI^04cb an additional three times in Vero-cCD150 cells and sequenced total RNA using our Illumina NGS pipeline. At P1, about 20% of the defective genome reads were non-rule-of-six copyback and deletion sequences, with only 1 sequencing read each (Fig. 4B, left axis). These numbers decreased drastically at P2 and P3, leaving nDI^RI^04cb as the predominant sequence, although the overall number of reads in the samples also dropped (Fig. 4B, right axis). This highlighted a production issue when we used a producer virus, due to the competition between the DI and full-length genome (Fig. 4C). To solve this issue, we first quantified the genome copies of nDI^RI^04cb or sDI^RI^LedTomTr present in P1 and the number of full-length genome copies in our producer stock using a quantitative RT-PCR (qRT-PCR) assay (Table 1). We then empirically determined that a low DI-to-virus ratio (0.05:1) repeatedly generated between 10^5^ and 10^7^ copies/ml of the DI genome (Fig. 4D), providing a way to control the DIP output.

### UV irradiation for targeted inactivation of producer virus.

**FIG 5:**
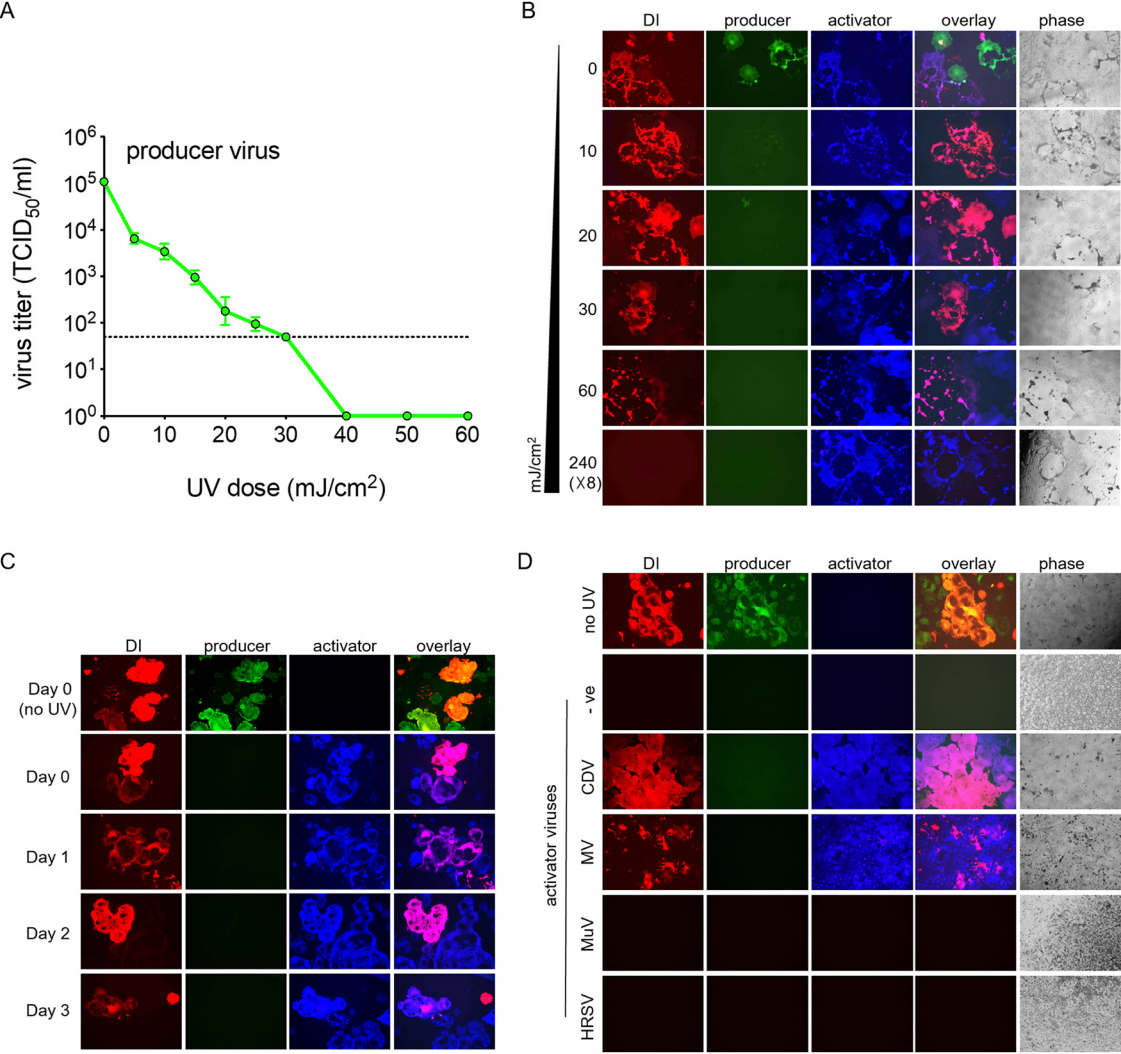
UV inactivation of producer virus and activation of DI with activator virus. (A) Effects of various UV dosages on rCDV^RI^ producer virus titers. Samples were irradiated using a UV crosslinker (CX-2000 crosslinker; UVP), and TCID_50_s per milliliter were determined. The dotted line represents the limit of detection of the assay. Error bars represent standard deviations (*n* = 3). (B) UV doses were tested in the presence of a DI genome (red) empirically to determine the required amount to inactivate the rCDV^RI^ producer virus and not the DI genome. Vero-cCD150 cells infected with UV-treated samples were superinfected with the rCDV^RI^TagBFP activator virus, in order to drive the replication of the DI genome in the samples. (C) The stability of the DI genome in infected cells was tested. DI genomes remained dormant in cells for up to 3 days, as shown by genome activation with rCDV^RI^. (D) Activation of the rCDV^RI^ DI genome using various paramyxoviruses as the activator virus. We tested the cross-reactivity of rCDV^RI^ DIP to other paramyxoviruses: measles virus (MV), mumps virus (MuV), and human respiratory syncytial virus (HRSV). Activation of an rCDV^RI^ DI genome occurred only with CDV and MV, both morbilliviruses.

To assess defective genome-specific effects without the confounding effects of the producer virus, we first tested various UV dosages that would sufficiently inactivate only the full-length genome. Briefly, producer virus samples were irradiated using a UV cross-linker at UV dosages between 0 and 60 mJ/cm^2^ (2 ml in a 6-well tray). By 40 mJ/cm^2^, no infectious virus could be detected via a 50% tissue culture infective dose (TCID_50_) assay (Fig. 5A).

Next, we tested these UV dosages in our DIP stocks in order to determine if the DI genome remained active. Since an active full-length genome is required for defective genome replication, we superinfected Vero-cCD150 cells that were infected with a range of UV-treated DIP stocks. Here, we used rCDV^RI^TagBFP(6) as an activator virus (blue syncytia in Fig. 5B and C). We found that the defective genome (red syncytia in Fig. 5B) remained active even at 60 mJ/cm^2^. In the subsequent experiments, we therefore chose 60 mJ/cm^2^ for producer virus inactivation. For complete inactivation of the DI genome, we used a high UV dose of 240 (× 8) mJ/cm^2^, as a single dose proved insufficient. Interestingly, we also found that defective genomes can remain inactive in cells for at least 3 days postinfection until superinfected with an activator virus (Fig. 5C).

Next, to confirm whether defective genome activation was specifically due to the activator virus and/or whether other paramyxoviruses can replicate the defective genome, we infected Vero-cCD150 cells with DIPs and then attempted to activate expression using CDV, measles virus (MV), mumps virus (MuV), or HRSV. Activation of a defective CDV genome occurred with CDV and MV but not with MuV or HRSV (Fig. 5D).

### rCDV^RI^ DIP-specific interference activity is dose dependent.

**FIG 6:**
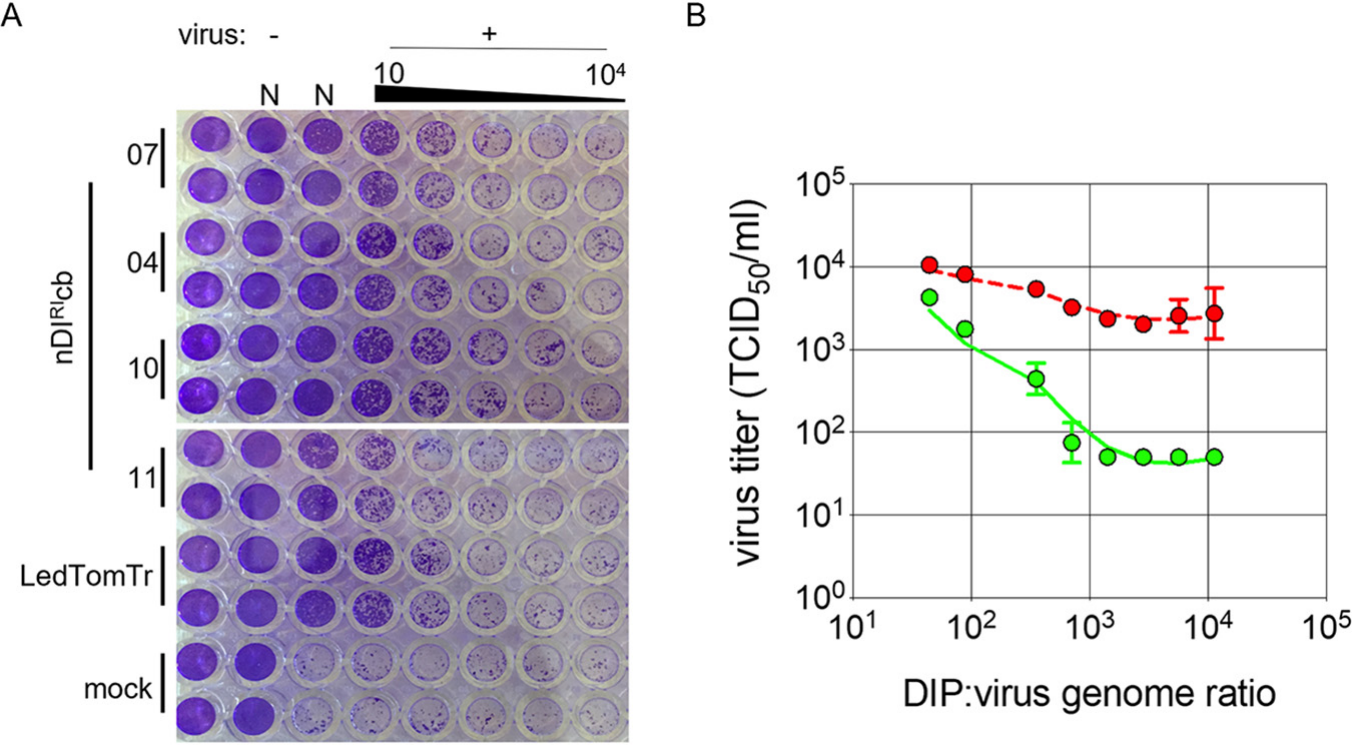
DIP interference on rCDV^RI^ infection *in vitro*. (A) Natural copyback DIs (nDIs) 07, 04, 10, and 11 (Table 2) and synthetic deletion DIP sDI^RI^LedTomTr were tested for their ability to interfere with rCDV^RI^ replication *in vitro*. The amount of inhibition of rCDV^RI^ infection in Vero-cCD150 cells is visualized by cytopathic effect (CPE) (cell monolayers stained with crystal violet). N, neat, undiluted DIP stock. (B) Dose-response curve for copyback sequence nDI^RI^04cb. To obtain a dose at which a DIP completely eliminates CDV infection, Vero-cCD150 cells were treated with specific ratios of virus and DIPs (green line). Control infections were performed using UV-inactivated DIPs (red line) to demonstrate that interfering effects were DI genome replication specific. rCDV^RI^ titers were measured by determining the TCID_50_ per milliliter at 72 h postinfection. Error bars represent standard deviations (*n* = 3).

We tested four cbDIPs (nDI^RI^04cb, nDI^RI^07cb, nDI^RI^10cb, and nDI^RI^11cb) (Table 2) and sDI^RI^LedTomTr for their ability to interfere with rCDV^RI^ replication *in vitro* using Vero-cCD150 cells, which lack a fully functional IFN system. Vero-cCD150 cell monolayers infected with a 10-fold serial dilution of the DIP stocks demonstrated almost complete inhibition of rCDV^RI^ infection at the highest DIP concentration (Fig. 6A). Using cbDIP nDI^RI^04cb, we determined if inhibition was DIP specific or an effect due to cytokines and/or cell debris. DIPs were UV inactivated at 120 (× 8) mJ/cm^2^ to ensure complete DI genome inactivation. Next, Vero-cCD150 cells were infected with various ratios of rCDV^RI^ and active DIPs or rCDV^RI^ and inactivated DIPs. Ratios were based on genome copies determined using a qRT-PCR assay. We found a dose-dependent DIP-specific interference effect in which a genome ratio of 10,000 DIPs to 1 rCDV^RI^ genome was required to completely inhibit virus replication in Vero-cCD150 cells (Fig. 6B). Importantly, we observed no reduction in rCDV^RI^ yield with the inactivated DIPs, demonstrating that the effect was DIP specific and not a result of cytokines/debris within the DIP inoculum.

### rCDV^RI^ DIPs replicate in appropriate cells in a ferret model.

**FIG 7:**
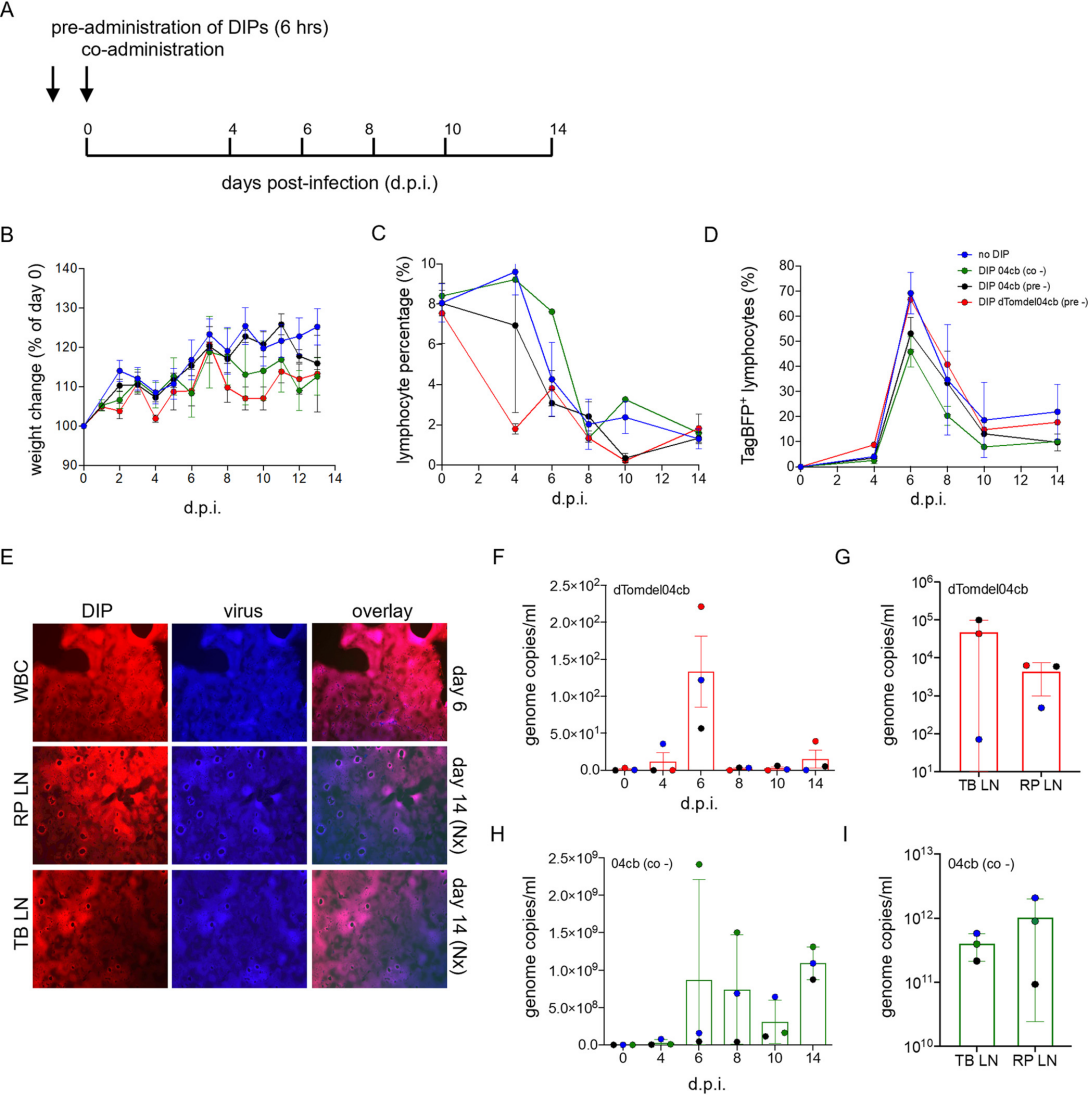
rCDV^RI^ infection in ferrets. (A) Experimental outline of ferret infection. Ferrets were either pre- or coadministered with DIP nDI^RI^04cb and preadministered with sDI^RI^dTomdel04cb or culture medium prior to infection with the challenge virus rCDV^RI^TagBFP. Preadministration was carried out 6 h before infection. (B) Weight change of ferrets during the course of infection. (C) Lymphocyte percentage in EDTA-blood determined by flow cytometry show lymphopenia. (D) Virus infection in lymphocytes was determined for each time point by monitoring fluorescence using flow cytometry. A peak in infection is seen at day 6. (E) sDI^RI^dTom04cb and virus were isolated from WBC (6 dpi) and from lymph nodes (14 dpi) from ferrets. TB-LN, tracheal-bronchial lymph node; RP-LN, retropharyngeal lymph node. (F and G) sDI^RI^dTom04cb genome copies were detected in WBC at each time point during the course of the infection and at necropsy in single-cell suspensions of lymphoid tissues. (H and I) nDI^RI^04cb genome copies were detected in WBC at each time point during the course of the infection and at necropsy in lymphoid tissues.

To assess if DIP nDI^RI^04cb can replicate and be maintained during the course of infection in a natural host animal model, four groups of three ferrets were infected with rCDV^RI^TagBFP(6) as an activator virus. Animals were either coadministered nDI^RI^04cb or Dulbecco’s modified Eagle’s medium (DMEM) (control) or preadministered nDI^RI^04cb or sDI^RI^dTomdel04cb (nDI^RI^04cb variant expressing dTom). DIPs were preadministered 6 h prior to activator virus infection. All infections were carried out via the intratracheal (IT) route (Fig. 7A). Animals were monitored over a 14-day period for clinical signs and symptoms, and blood samples were collected at 0 days postinfection and then every 2 days. No weight loss was observed (Fig. 7B) over the infection period. All animals had lymphopenia, (Fig. 7C) which is typical in ferrets infected with CDV. White blood cells (WBC) were isolated from the blood samples, and the percentage of TagBFP-positive cells was determined by flow cytometry (Fig. 7D); this was confirmed at the same time by virus isolation in Vero-cCD150 cells. rCDV^RI^TagBFP(6) was detected in the WBC of all ferrets, with a peak at 6 dpi (Fig. 7E). At necropsy, rCDV^RI^TagBFP(6) was also isolated from the lymph nodes of all animals. Importantly, we isolated sDI^RI^dTomdel04cb from the WBC of two animals at 6 dpi and the lymph nodes at 14 dpi (Fig. 7E). Using a genome-specific and DI-specific qRT-PCR assay, we detected low levels of DI genomes in the WBC and lymph nodes for both sDI^RI^dTomdel04cb (Fig. 7F and G) and nDI^RI^04cb (Fig. 7H and I).

## DISCUSSION

Viruses adapt quickly to their host environment, and so it is crucial to minimize their passage *in vitro* if we are to obtain meaningful information. In the present study, we describe the establishment of a novel recombinant canine distemper virus strain from Rhode Island (rCDV^RI^). rCDV^RI^ is the first non-laboratory-adapted rCDV, based on a currently circulating wild-type strain isolated from a raccoon in Rhode Island, USA, in 2012. We propose that rCDV^RI^ should replace all currently used laboratory-adapted rCDV strains, such as Snyder-Hill and A75, in pathogenesis studies. The accessibility and affordability of sequencing and synthetic biology should now allow the use of field isolates of known provenance, such as CDV^RI^, to replace laboratory-adapted strains.

The overarching goal of this study was to assess the ability of a non-laboratory-adapted CDV to generate DI genomes *in vitro* and whether these DI genomes could be maintained *in vivo* in a natural animal model. We used a combination of RT-PCR/Sanger sequencing and short-read Illumina sequencing to identify defective genomes in rCDV^RI^. We used a bioinformatics pipeline (31) to identify DI junctions in the Illumina data sets and found that all high-frequency copyback DI junctions that were identified matched those isolated by RT-PCR. Our results revealed that defective genomes are generated early during rCDV^RI^ rescue and passage. Independently rescued rCDV^RI^ stocks contained unique defective genomes, out of which one predominant copyback genome persisted alongside the full-length genome during serial passage. We were unable to detect any deletion genomes by RT-PCR, potentially due to their low abundance, as revealed in the Illumina data set. However, it should be noted that frequency of a DI junction does not reflect the true quantity of a genome in the sample. Further, as morbillivirus genomes are multiples of six, we expect that only rule-of-six-compliant defective genomes successfully compete with the full-length genome. All high-frequency copyback DI genomes that we identified were indeed multiples of six. Recent data on HRSV suggests that DI genome generation may be sequence driven rather than a random process (14), we did not find this to be the case in our studies on rCDV^RI^. Different viruses potentially have different processes for generating/regulating DI genomes; for instance, deleting the C protein from some paramyxoviruses increases DI formation (32–34).

Next, we combined the use of an attenuated rCDV^RI^ as the producer virus with selective UV inactivation to address DIP production and purification. We successfully demonstrated that such DIPs can be maintained during the course of rCDV^RI^ infection in ferrets. If DIPs are to be used therapeutically, then obtaining an appropriate DIP dosage is essential. However, translating dosage from *in vitro* to *in vivo* can be challenging. Our *in vitro* dose-response assay for copyback DIP nDI^RI^04cb determined a DIP-to-virus ratio at about 10,000:1 (Fig. 6B). Similar dose responses were observed for Nipah virus (31). However, infecting ferrets with a high dose of 70,000 DI genomes to 1 virus genome proved inadequate to change the course of infection in these animals. From our experience, using the deletion DIP sDI^RI^LedTomTr, we found that DI genome copies do not necessarily translate to infectious DIP titers (unpublished data). Therefore, our true dose may have been well below that required for an *in vivo* system. Our *in vitro* assays using IFN-deficient Vero-cCD150 cells demonstrate that DIPs can inhibit wild-type virus generation. Nevertheless, we believe that any *in vivo* effect using these DIPs or any DIP-based therapy may ultimately be down to innate immune responses mounted by the host.

DIP production is a work in progress and will be an important hurdle to decipher for DIP-based therapeutics. We chose an attenuated producer virus approach for this study. rCDV^RI^ producer virus was based on our previous work demonstrating that introduction of foreign sequences in the second variable hinge of the L protein sufficiently attenuates morbilliviruses (29, 30). As expected, rCDV^RI^Venus(6)L_EGFP_ was also attenuated in ferrets (unpublished data). This is important, as such a producer virus would not contribute to disease, thus making it possible to assess a potential interference effect of the DIP. DIPs generated with such a system will be properly packaged and delivered to the right target cells. Additionally, UV inactivation of such a producer virus would no longer be a safety concern. However, obtaining the high DIP concentrations required using such a system is still a tedious task, and any shift in balance results in reduced DIP titers.

DIPs may well have a future as therapeutic interfering particles, but before we can safely make that transition, we need to consider the long-term effects of DIPs, such as their role in viral persistence (35). Extensive longitudinal experiments in appropriate animal models would need to be carried out to address any safety issues. Although the DIPs in this study were (at our estimated ratio) unable to interfere with wild-type CDV, the study provides formal evidence for sustained DI genome replication *in vivo* and provides a valuable basis for future DIP work with (−) RNA viruses.

## MATERIALS AND METHODS

### Cell lines, plasmids, and viruses.

Vero cells stably expressing canine receptor CD150 (Vero-cCD150 cells) were grown in advanced Dulbecco’s modified Eagle’s medium (DMEM; Gibco) supplemented with 10% (vol/vol) heat-inactivated fetal bovine serum (HI FBS; Gibco) and GlutaMAX-I (Gibco) (36). Cells were grown at 37°C and 5% CO_2_.

CDV was isolated from raccoon oral swabs, and viral consensus sequences were obtained (GenBank accession number KU666057). To construct a plasmid containing the full-length genome, we obtained seven synthetic fragments with overlapping sequences and assembled them via Gibson assembly (NEBuilder HiFi DNA assembly; NEB). A subclone containing the remainder of the viral sequences and restriction sites required to clone in the fragments was generated in a modified pBluescript plasmid (38). The Gibson assembly fragments were then cloned into the subclone, generating pCDV^RI^.

pCDV^RI^ plasmids expressing Venus, dTom, TagBFP, or Gluc were generated by placing the coding sequences as an additional transcription unit at position six in the genome. Briefly, the parental plasmid (pCDV^RI^) was linearized using the restriction sites MseI at genome position 8340 (H gene) and AatII at genome position 10696 (L gene); reporter genes (obtained as gBlock gene fragments from IDT) were then ligated into the linearized plasmid by Gibson assembly (NEBuilder HiFi DNA assembly; NEB). The phosphoprotein gene start and the hemagglutinin (H) glycoprotein gene end sequences were used as signal sequences for the reporter genes. pCDV^RI^Venus(6)-L_EGFP_ was constructed from pCDV^RI^Venus(6). We used restriction site AvrII to linearize pCDV^RI^Venus(6) at genome positions 14141 and 15060. We then used a 1,690-bp synthetic fragment (gBlock gene fragments from IDT) containing the digested L gene sequences and EGFP to be inserted by Gibson assembly (NEBuilder HiFi DNA assembly; NEB). The gBlock contained mutations to replace the CT nucleotides at rCDV^RI^Venus(6) genome positions 14826-27 and 14835-36 with GG and AA, respectively. These mutations create the restriction sites MscI and HpaI in pCDV^RI^Venus(6)-L_EGFP_, which allows EGFP to be swapped easily with other genes when needed.

The expression plasmids encoding the N, P, and L protein sequences of CDV^RI^ were generated by amplifying the genes from pCDV^RI^ by PCR. The PCR fragments were then ligated into the eukaryotic expression vector pCG (39) using AscI and SpeI restriction sites, to generate pCG-CDV^RI^N and pCG-CDV^RI^P, and AscI and AfeI restriction sites, to generate pCG-CDV^RI^L.

As with the virus rescue plasmids, all defective-genome-expressing plasmids were T7-driven. Defective genomes from the T7 transcripts were designed to be in the negative-sense orientation. First, a copyback genome core plasmid containing 22 nucleotides of trailer (AGP) and trailer complement sequences with a naturally formed EcoRV restriction site was generated. This allowed cassettes (gBlock gene fragments from IDT) containing various copyback genomes to be ligated using Gibson assembly (NEBuilder HiFi DNA assembly; NEB). The deletion genome core plasmid was designed to contain EcoRV and StuI restriction sites in the trailer and leader (GP) sequences, respectively. As with the copyback plasmids, this allows various deletion genome cassettes to be easily ligated using Gibson assembly. All plasmids described here were sequence verified using Sanger sequencing (Genewiz; USA).

Viruses rCDV^RI^Venus(6), rCDV^RI^dTom(6), rCDV^RI^TagBFP(6), rCDV^RI^Gluc(6), and rCDV^RI^Venus(6)-L_EGFP_ were rescued, grown, and titrated in Vero-cCD150 cells by infecting the cells with MVA-T7 for 1 h at 37°C. Inoculum was aspirated, and cells were transfected (Lipofectamine 2000; Life Technologies) with pCG-CDV^RI^N, pCG-CDV^RI^P, pCG-CDV^RI^L, and pCDV^RI^Venus(6), pCDV^RI^dTom(6), pCDV^RI^TagBFP(6), pCDV^RI^Gluc(6), or rCDV^RI^Venus(6)-L_EGFP_. After 18 h, the transfection mix was removed and replaced with complete growth medium. Cells were incubated for up to 5 to 7 days at 37°C with 5% (vol/vol) CO_2_. The presence of virus was confirmed by observing cytopathic effects (CPEs) by phase-contrast microscopy and fluorescence microscopy. Virus stocks were grown on Vero-cCD150 cells and subjected to one freeze-thaw cycle, and debris was removed by centrifugation at 3,000 rpm for 10 min at 4°C. The cleared supernatant (virus stock) was aliquoted and titrated in Vero-cCD150 cells; calculated quantities, expressed in TCID_50_ units, were used to calculate MOIs for infections.

### Virus passage.

Vero-cCD150 cell monolayers at 2 × 10^5^ cells/ml in T25 flasks were infected with rCDV^RI^Venus(6), rCDV^RI^TagBFP(6), rCDV^RI^dTom(6), or rCDV^RI^Gluc(6) at an MOI of 0.05. At 72 h postinfection, cells were scraped into culture medium and placed at −80°C. After freeze-thawing, cell debris were clarified, and 2 ml of this was used to infect fresh Vero-cCD150 cells in a T25 flask, and so on. Viral titers were determined as TCID_50_/ml for each passage.

### Copyback genome identification by Sanger sequencing.

Total RNA was extracted using TRIzol LS reagent (ThermoFisher) according to the manufacturer’s recommendations, and the RNA pellet was resuspended in 40 μl nuclease-free water (Invitrogen). cDNA was generated with 7 μl of resuspended RNA using the SuperScript III first-strand synthesis system (Thermo Fisher Scientific) and primers A1 and A2 (Table 1) in a total volume of 20 μl. Two microliters of the resultant cDNA was then amplified with primers for copyback genome (A1 and A2) and L gene (A1 and B1) amplification (Table 1), using Phusion high-fidelity DNA polymerase (NEB) in a total volume of 50 μl (using a touchdown PCR amplification protocol). PCR products were analyzed on a 1% agarose gel and bands gel purified using a QIAquick gel extraction kit (Qiagen). Samples were sequenced via Sanger sequencing (Genewiz; USA).

### Defective genome identification by Illumina sequencing.

Total RNA was extracted using TRIzol LS reagent (ThermoFisher) according to the manufacturer’s recommendations. The RNA pellet was resuspended in 40 μl nuclease-free water (Invitrogen). Library preparation, rRNA depletion, and sequencing were carried out according to manufacturer recommendations for the MiniSeq system (Illumina). Illumina reads were trimmed of their adaptor sequences, quality checked using an in-house script, and then mapped to the appropriate reference genome with bwa-mem using default parameters (version 0.7.7). With a modified version (31) of script chimeric_reads.py v3.6.2 (40, 41), chimeric reads were identified among each data set.

### DIP rescue, production, and quantification.

Vero-cCD150 cell monolayers in 6-well trays were infected with Fowlpox-T7 in Opti-MEM (Gibco) for 30 min at 37°C and then spinoculated at room temperature for another 30 min. Medium was then removed, and cells were transfected with 2 μg of defective-genome-expressing plasmid, along with pCG-CDV^RI^-N, pCG-CDV^RI^-P, and pCG-CDV^RI^-L for 48 h. Transfected cells were then superinfected with rCDV^RI^Venus(6)-L_EGFP_ at an MOI of 0.001. Two days postinfection, cells were trypsinized and passaged into a T75 flask. Cells were harvested by freeze-thawing when CPEs were visible. rCDV^RI^Venus(6)-L_EGFP_ producer virus was inactivated from all DIP stocks at 60 mJ/cm^2^ in a 2-ml culture volume in a 6-well tray using a CX-2000 crosslinker (UVP).

For quantification, total RNA was extracted using TRIzol LS reagent (ThermoFisher) according to the manufacturer’s recommendations, and the RNA pellet was resuspended in 40 μl nuclease-free water (Invitrogen). Three microliters of extracted RNA was used in a TaqMan one-step qRT-PCR assay (Luna Universal one-step RT-qPCR kit; NEB). TaqMan analysis was carried out with the primer/probe combination described in Table 1, and analysis was performed on the QuantStudio 6 flex system (ThermoFisher Scientific).

### DI interference assay.

DIP stocks were split in half and UV irradiated to inactivate either the producer virus (60 mJ/cm^2^, active DIPs) or the DIPs (120 mJ/cm^2^ [× 8], inactive DIPs). UV irradiation was carried out in 2 ml in a 6-well tray using a CX-2000 crosslinker (UVP). Vero-cCD150 cell monolayers in 24- or 96-well trays were infected with either active or inactive DIPs for 1 h at 37°C and then superinfected with rCDV^RI^ for 1 h at 37°C. The supernatant was then removed, and cells were washed with phosphate-buffered saline (PBS) 3 times. Fresh medium was added onto the cells, and they were incubated for 48 h. For crystal violet staining, cells were fixed in formalin for 15 min and then stained with crystal violet for another 15 min. Cells were washed with water and visualized. For virus titers, cells and medium were freeze-thawed at −80°C and TCID_50_ determinations carried out on Vero-cCD150 cells.

### Animal study design.

The animal experiment described here was conducted in compliance with all applicable U.S. Government policies and regulations and AAALAC international standards for the humane care and use of animals. All protocols were approved by the Boston University institutional animal care and use committee. Twelve 16-week-old CDV-seronegative male ferrets (Mustela putorius furo) were housed in groups of three. The cages contained toys as a source of environmental enrichment. Animals were intratracheally (IT) inoculated with 10^4^ TCID_50_s of rCDV^RI^TagBFP(6). Nine ferrets, divided into groups of three, were also inoculated with DIPs. One group was inoculated with DIPs 6 h prior to rCDV^RI^TagBFP(6). Inoculation was carried out with 2 ml of DIPs or medium and 40 μl of the rCDV^RI^TagBFP(6) suspension. Animals were monitored several times per day, and blood samples were collected every 2 days postinoculation in Vacuette tubes containing EDTA as an anticoagulant. Procedures were performed under light anesthesia (initial sedation with ketamine, medetomidine, and butorphanol followed by maintenance with 1 to 5% isoflurane in oxygen and atipamazole reversal after handling). All animals were euthanized 14 days postinoculation, and necropsies were performed.

### Sample processing and analysis.

Fifty microliters of a blood sample was analyzed on a VetScan HM5 apparatus (Abaxis) to determine total WBC and lymphocyte counts. Red blood cells (RBC) were then lysed using 1× multispecies RBC lysis buffer (eBioscience). WBC were washed in Dulbecco’s PBS (D-PBS), collected by centrifugation (350 × *g*, 10 min), and resuspended in 300 to 500 μl D-PBS. The resuspended WBC were used to determine total lymphocyte counts and infected-WBC percentages by flow cytometry using an LSRII flow cytometer (BD LSR II; BD Biosciences). Virus isolation from WBC was performed on Vero-cCD150 cells by titrating the WBC in Opti-MEM.

Ferrets were euthanized by administration of an overdose of barbiturate anesthetic under deep sedation. Lymphoid tissues were collected during necropsy and placed in PBS for single-cell suspensions. Fatty tissue was removed from the lymphoid tissues before the tissues were dissected into smaller pieces to allow dissociation using gentleMACS dissociation (C) tubes (Miltenyi Biotec) in advanced RPMI medium supplemented with 10% fetal calf serum, 1% GlutaMAX, and 1× antibiotic-antimycotic (ThermoFisher Scientific). Samples were dissociated on a gentleMACS dissociator (Miltenyi Biotec) using the m_spleen_C preset parameter. Samples were then passed through cell strainers with a 100-μm pore size (Falcon cell strainers), washed in D-PBS, and pelleted by centrifugation (350 × *g*, 10 min). Pellets were resuspended in an appropriate volume of D-PBS and analyzed by flow cytometry as described above.
